# Severe influenza infection is associated with inflammatory programmed cell death in infected macrophages

**DOI:** 10.3389/fcimb.2023.1067285

**Published:** 2023-02-16

**Authors:** André C. Ferreira, Carolina Q. Sacramento, Filipe S. Pereira-Dutra, Natália Fintelman-Rodrigues, Priscila P. Silva, Mayara Mattos, Caroline S. de Freitas, Andressa Marttorelli, Gabrielle R. de Melo, Mariana M. Campos, Isaclaudia G. Azevedo-Quintanilha, Aluana S. Carlos, João Vítor Emídio, Cristiana C. Garcia, Patrícia T. Bozza, Fernando A. Bozza, Thiago M. L. Souza

**Affiliations:** ^1^ Laboratory of Immunopharmacology, Oswaldo Cruz Institute, Fundação Oswado Cruz (Fiocruz), Rio de Janeiro, RJ, Brazil; ^2^ National Institute for Science and Technology on Innovation on Neglected Diseases (INCT/IDN), Center for Technological Development in Health (CDTS), Fundação Oswado Cruz (Fiocruz), Rio de Janeiro, RJ, Brazil; ^3^ Preclinical Research Laboratory, Universidade Iguaçu (UNIG), Nova Iguaçu, RJ, Brazil; ^4^ Respiratory and Measles Virus Laboratory, Oswaldo Cruz Institute, Fundação Oswado Cruz (Fiocruz), Rio de Janeiro, RJ, Brazil; ^5^ National Institute of Infectious Disease Evandro Chagas, Fundação Oswado Cruz (Fiocruz), Rio de Janeiro, RJ, Brazil; ^6^ Department of Critical Care, Instituto D’Or de Pesquisa e Ensino (IDOR), Rio de Janeiro, RJ, Brazil

**Keywords:** influenza virus, necroptosis, TNF, etanercept, macrophages

## Abstract

**Introduction:**

Influenza A virus (IAV) is one of the leading causes of respiratory tract infections in humans, representing a major public health concern. The various types of cell death have a crucial role in IAV pathogenesis because this virus may trigger both apoptosis and necroptosis in airway epithelial cells in parallel. Macrophages play an important role in the clearance of virus particles, priming the adaptive immune response in influenza. However, the contribution of macrophage death to pathogenesis of IAV infection remains unclear.

**Methods:**

In this work, we investigated IAV-induced macrophage death, along with potential therapeutic intervention. We conducted in vitro and in vivo experiments to evaluate the mechanism and the contribution of macrophages death to the inflammatory response induced by IAV infection.

**Results:**

We found that IAV or its surface glycoprotein hemagglutinin (HA) triggers inflammatory programmed cell death in human and murine macrophages in a Toll-like receptor-4 (TLR4)- and TNF-dependent manner. Anti-TNF treatment in vivo with the clinically approved drug etanercept prevented the engagement of the necroptotic loop and mouse mortality. Etanercept impaired the IAV-induced proinflammatory cytokine storm and lung injury.

**Conclusion:**

In summary, we demonstrated a positive feedback loop of events that led to necroptosis and exacerbated inflammation in IAV-infected macrophages. Our results highlight an additional mechanism involved in severe influenza that could be attenuated with clinically available therapies.

## Introduction

Acute respiratory illness caused by influenza A (IAV) is associated with epidemic and pandemic outbreaks (Webster et al., 1992). Influenza presentation ranges from mild to potentially lethal cases of severe acute respiratory syndrome (SARS) (Paules and Subbarao, 2017; World Health Organization, 2019). Annually, there are an estimated 1 billion cases of influenza, of which 3–5 million are severe cases and 650 thousand are influenza-associated deaths from respiratory causes alone (Iuliano et al., 2018; Paget et al., 2019; World Health Organization, 2019). Furthermore, influenza viruses are a dangerous threat to global public health systems, causing occasional pandemic outbreaks more recently during 2009 (Fineberg, 2014).

IAV has a negative-sense-segmented RNA genome coding for at least 11 viral proteins, of which hemagglutinin (HA) and neuraminidase (NA) are the major surface glycoproteins (Resa-Infante et al., 2011). During its life cycle, HA attaches to sialic acid residues on the cellular surface, allowing IAV to be endocytosed (Kalil and Thomas, 2019). After HA-dependent fusion of the viral envelope with the endosome membrane, viral RNA and associated proteins are transported to the cellular nucleus, where replication and transcription take place. Newly synthesized proteins are assembled, virions bud the cell membrane, and NA assists in their final release (Yatim and Albert, 2011). During this process, infected cells may succumb to death. The various fates of cell death have an integral role in IAV pathogenesis (Yatim and Albert, 2011; Nailwal and Chan, 2019). Recent data reported that IAV infection activates the receptor interaction serine/threonine-protein kinases 1 and 3 (RIPK1 and RIPK3), promoting both apoptosis and necroptosis in airway epithelial cells (Kuriakose et al., 2016; Nogusa et al., 2016; Thapa et al., 2016; Zhang et al., 2020). Apoptosis has been proposed as cell death related to immune tolerance to IAV infection, a host defense mechanism limiting viral immunopathology (Atkin-Smith et al., 2018). IAV-induced necrotic cell death was reported to be crucial in antiviral host defense, with inflammatory engagement (Shubina et al., 2020; Zhang et al., 2020). During necroptosis, cells are triggered to shift from apoptotic-like cell death to disruption of the plasma membrane, facilitating the release of immunomodulatory damage associated with molecular patterns (DAMPs) and leading to inflammation (Orzalli and Kagan, 2017). The proinflammatory cytokine storm is an important hallmark of IAV-induced severe pneumonia and is strongly associated with lung/respiratory dysfunction (Yang and Tang, 2016).

Moreover, IAV physiopathology is not restricted to airway epithelial cells because macrophages are also exposed to this virus. During severe influenza, blood monocytes migrate toward the respiratory tract (Sakabe et al., 2011; Tisoncik et al., 2012), where they differentiate into macrophages. Macrophages play an essential role in the clearance of virus particles and priming the adaptive immune response (Meischel et al., 2020). IAV-infected macrophage death has been related to overproduction of local and systemic cytokines/chemokines, cellular infiltration, extracellular matrix degradation, and airway epithelial denudation (Rodrigue-Gervais et al., 2014).

Some evidence suggests a link between necroptosis and exacerbation of the inflammatory response during IAV pathogenesis (Rodrigue-Gervais et al., 2014; Nailwal and Chan, 2019; Zhang et al., 2020). However, the contribution of macrophage death to the inflammatory response and pathogenesis of IAV infection remains unclear. In this work, we investigated IAV-induced macrophage death. Our findings indicate that IAV- and HA-induced inflammatory programmed cell death in primary macrophages leads to a loop of events involving Toll-like receptor 4 (TLR4), tumor necrosis factor (TNF), and RIPK1, resulting in exacerbation of the inflammatory response. Moreover, we show that the blockade of TNF-related cell death signaling by the biodrug etanercept increased the survival of IAV-infected animals and jeopardized the inflammatory response associated with severe IAV infections.

## Materials and methods

### Reagents

We used different pharmacological inhibitors throughout this study (**Table 1**). The concentration of each inhibitor was chosen based on the manufacturer’s recommendations. All inhibitors were dissolved in 100% dimethylsulfoxide (DMSO) and subsequently diluted at least 10^4^-fold in culture medium before each assay. The final DMSO concentrations showed no cytotoxicity.

**Table 1:** List of small molecules and biodrugs used as pharmacological inhibitors.

Compound	Abbreviation	Target	Concentration	Manufacture	Catalog code
Z-VAD-FMK	ZVAD	Pancaspase	10 µM	*In vivo*gen	tlrl-vad
Necrostatin-1	Nec-1	RIPK1	25 µM	Sigma-Andrich	64419-92-7
Recombinant Mouse TNF-alpha	TNF	TNF-receptor	1 ng/mL	R&D System	410-MT/CF
Resatorvid	CLI95	TLR4	2 µM	*In vivo*gen	tlrl-cli95
anti-TNF antibody	anti-TNF	TNF	1 ng/mL	R&D System	MAB4101
Polymyxin b	POLI	LPS	100 ng/mL	Sigma−Aldrich	P4932
Lipopolysaccharide serotype 0111: B4	LPS	TLR4	10 ng/mL	Sigma−Aldrich	L4931
Etanercept	ETN	TNF-receptor	2.5 mg/kg	Pfizer	ENBREL^®^

In addition to the pharmacological inhibitors, we used recombinant influenza A virus hemagglutinin H1 protein (HA, Abcam, Cat.# ab217651) from a HEK 293 cell expression system. To select the HA dose, macrophages were exposed to different concentrations of this protein or lipopolysaccharide (LPS; Sigma Aldrich). We observed that 10 ng/mL HA was the minimal dose to induce TNF (**Figure S1**), and 10 ng/mL LPS was used as a positive control (**Figure S1**). In all experiments with HA, polymyxin B was used to neutralize possible contaminating LPS.

### Virus strain and growth conditions

Madin-Darby canine kidney (MDCK) cells were cultured in Dulbecco’s modified Eagle’s medium (DMEM; Life Technologies) supplemented with 10% fetal bovine serum (FBS; HyClone), 100 U/mL penicillin, and 100 mg/mL streptomycin (Sigma−Aldrich) at 37°C in a 5% CO_2_ atmosphere.

IAV, A/H1N1/Puerto Rico/8/34 strain (PR8), was grown and titrated in MDCK (Szretter et al., 2006; World Health Organization, 2011). Briefly, confluent cells in 75 cm^2^ culture flasks were infected with PR8 at a multiplicity of infection (MOI) of 0.1. The inoculum was prepared in DMEM containing 2 µg/ml TPCK-trypsin (Sigma−Aldrich). Cells were exposed to PR8 for 1 h at 37°C to allow virus adsorption. After the incubation period, the cells were washed with PBS, and fresh inoculation medium was added. Cultures were observed daily until the detection of IAV-induced cytopathic effects (CPEs). Culture supernatants containing the viruses were collected and centrifuged (1,500 rpm for 5 minutes) to remove cell debris. The viral stocks were aliquoted and stored at -70°C for further studies.

Titration was performed through plaque assays using 10-fold dilutions (10^-1^ to 10^-10^) in 6-well plates. After 1 h of incubation with virus dilutions, the inoculum was removed, the cells were washed, and fresh DMEM with 2.5% agarose with TPCK-trypsin at 1 µg/ml was added. After 3 days at 37°C, the agar was removed, and the wells were washed with PBS and stained with crystal violet 0.1%.

### Mice

C57BL/6 mice (20–30 g) were supplied by the Institute of Science and Technology in Biomodels from Oswaldo Cruz Foundation and used at 8–12 weeks of age. The Institutional Animal Welfare Committee (Committee on the Use of Laboratory Animals of the Oswaldo Cruz Foundation) approved all animal experiments in agreement with the Brazilian National guidelines supported by CONCEA (Conselho Nacional de Controle em Experimentação Animal) under license number L050/15 (CEUA/FIOCRUZ). Mice were maintained with rodent diet and water available *ad libitum* with a 12 h light-dark cycle under controlled temperature (23 ± 1 °C).

### Murine and human macrophages

To obtain murine bone marrow-derived macrophages (BMDMs), cells were isolated from the femurs and tibias of mice on a C57BL/6 background, either from wild-type (WT), Toll-like Receptor 2 or 4 knockout (TLR2^-/-^ or TLR4^-/-^, respectively) mice. The isolated cells were cultured for 7 days in RPMI-1640 medium supplemented with 30% (vol/vol) L929 supernatant, 20% (vol/vol) heat-inactivated fetal bovine serum, 1% L-glutamine (vol/vol), and 1% penicillin−streptomycin (vol/vol) as previously described (Assunção et al., 2017). Differentiated macrophages were cultured in RPMI-1640 supplemented with 10% heat-inactivated fetal bovine serum (vol/vol), 1% L-glutamine (vol/vol), and 1% penicillin/streptomycin (vol/vol). BMDMs were maintained at a density of 5x10^5^ cells/ml.

Human monocyte-derived macrophages (MDMs) were obtained through plastic adherence of peripheral blood mononuclear cells (PBMCs). In brief, PBMCs were obtained from buffy coat preparations of healthy donors by density gradient centrifugation (Ficoll-Paque, GE Healthcare). PBMCs (2.0 x 10^6^ cells) were plated onto 48-well plates (NalgeNunc) in DMEM containing 10% human serum (HS; Millipore) and penicillin/streptomycin. Cells were maintained for monocyte differentiation into macrophages under standard culture conditions for 6–7 days. Then, nonadherent cells were washed, and the remaining macrophage layer was maintained in DMEM with 5% HS (Mesquita et al., 2014).

The purity of murine and human macrophages was above 95%, as determined by flow cytometric analysis (FACScan; Becton Dickinson) using anti-CD3 (BD Biosciences) and anti-CD16 (Southern Biotech) monoclonal antibodies.

### *In vitro* experiments

Murine or human macrophages were plated (5 x 10^5^ cells/well) in 24-well culture plates (flat-bottom, tissue-culture-treated plates; Costar) and incubated for 12 h at 37°C and 5% CO_2_. The cultures were then infected with PR8 (MOI of 0.25) or exposed to 10 ng/mL HA for 24 h. In parallel, as positive controls, macrophage cultures were also stimulated with 1 ng/mL TNF-α or 10 ng/mL LPS. After this incubation period, culture supernatants were collected for cell death analysis by LDH measurement and cytokine/chemokine quantification. The cell monolayers were harvested for flow cytometry and western blot analysis. To impair cell death, 10 μM zVAD, 25 μM Nec-1, 2 μM CLI95, or 1 ng/mL anti-TNF-α antibody was added to the cell culture and incubated for 30 min before IAV infection or the application of stimuli (HA, LPS or TNF) and remained for all infection/stimulus times at 37°C in 5% CO_2_.

### Analysis of cell death

Macrophage viability was evaluated by quantifying LDH and by flow cytometry in the presence of the pharmacological inhibitors. LDH quantification was performed after culture supernatant centrifugation (5,000 rpm for 1 minute) to remove cellular debris. According to the manufacturer’s instructions, extracellular lactate dehydrogenase (LDH) was quantified using the Doles^®^ kit. In summary, 25 µL of cell samples were seeded in 96-well culture plates and incubated with 5 µL of ferric alum and 100 µL of LDH substrate for 3 minutes at 37°C. Nicotinamide adenine dinucleotide (NAD, oxidized form) was added, followed by a stabilizing solution. After 10 minutes of incubation, plates were read in a spectrophotometer at 492 nm.

For flow cytometry analysis, macrophages were diluted in labeling buffer (10^6^ cells/mL). Then, 100 µL of cell samples were marked with 5 µL of Annexin V (BD Biosciences) and 1 µL of PI (BD Biosciences) for 15 minutes for cell death analysis. Approximately 10,000 events were acquired using a FACSCalibur, and analyses were performed using CellQuest software. Macrophages were gated through cell size (forward light scatter, FSC) and granularity (side light scatter, SSC) analysis (**Figure S2**). The profiles for macrophage positivity to AnnexinV and/or PI (AnnexinV^+^/PI^+^) were determined for cells from *in vitro* experiments and from BAL fluid of IAV-infected mice. Data acquisition was set to count a total of 10,000 events, and the FLOWJO software package was used to analyze the data.

### *In vivo* experiments

For infection procedures, mice were anesthetized with 60 mg/kg ketamine and 4 mg/kg xylazine and inoculated intranasally with PBS (MOCK) or 10^3^ PFU of PR8 in 25 µl of PBS (Marvin et al., 2017). The animals were kept under observation until they completely recovered. Six hours postinfection, the treated groups received an intraperitoneal dose of 2.5 mg/kg etanercept/ENBREL in 200 μL of vehicle (PBS). The treatment was continued with a daily dose of 2.5 mg/kg etanercept for 7 days. We used 10 mice per experimental group: mock-infected (MOCK); influenza-infected and treated with vehicle (IAV); and influenza-infected and treated with etanercept (IAV/ETN). The animals were monitored daily for 15 days for survival and eight days for body-weight analysis. In the case of weight loss higher than 25%, euthanasia was performed to alleviate animal suffering.

### Bronchoalveolar lavage and lung homogenates

Mice were euthanized on days 3 and 5 after infection to evaluate the lung inflammatory process induced by IAV infection. The mice were anesthetized, and bronchoalveolar lavage (BAL) from both lungs was harvested by washing the lungs three times with two 1-ml aliquots of cold PBS. After centrifugation of BAL (1500 rpm for 5 minutes), the pellet was used for total and differential leukocyte counts and cell death analysis by flow cytometry. The supernatant of the centrifuged BAL was used for cytokine/chemokine and total protein measurements and cell death analysis by LDH quantification. Total leukocytes (diluted in Turk’s 2% acetic acid solution) were counted using a Neubauer chamber. Differential cell counts were performed in cytospins (Cytospin3; centrifugation of 350 x *g* for 5 minutes at room temperature) and stained by the May-Grünwald-Giemsa method. The levels of cytokines and chemokines were assessed by ELISA. The total protein concentration in the BAL fluid was measured using a BCA protein assay kit (Thermo Scientific).

After BAL harvesting, the lungs were perfused with 5 ml of PBS to remove the circulating blood. Lungs were then collected and macerated in 750 µL of cold phosphate buffer containing protease inhibitor cocktail (Roche Applied Science, Mannheim, Germany). Homogenates were stored at −80°C for western blot analysis.

### Measurements inflammatory mediators

The levels of TNF, IL-6, IL-10, IFN-γ, CCL2 and CXCL1 were quantified in the *in vitro* macrophage supernatants and BAL fluid from IAV-infected mice using DuoSet^®^ ELISAs, following the manufacturer’s instructions (R&D Systems). Briefly, 100 µL of each sample was added to 96-well plates covered with the capture antibody. After a 2 h incubation period at room temperature (RT), the detection antibody was added, and the plates were incubated for a second round of 2 h at RT. Streptavidin-HRP and its substrate were added with a 20 min incubation interval, and the optical density was determined using a microplate reader set to 450 nm. Nitrite levels in cell-free culture supernatant were measured using the Griess reagent system according to the manufacturer’s instructions (Promega cat.# G2930).

### Western blot assay

Cellular extracts of 1x10^6^ cells or 0.5 g of tissue were homogenized in RIPA lysis buffer (1% Triton X-100, 2% SDS, 150 mM NaCl, 10 mM HEPES, 2 mM EDTA) containing protease and phosphatase inhibitor cocktail (Roche, pH 8.0). After centrifugation at 13 000 × g for 5 min, cell lysates were prepared under reducing and denaturing conditions and subjected to SDS−PAGE. Equal concentrations of proteins were fractionated by electrophoresis on 10% acrylamide gels. The proteins were transferred onto a nitrocellulose membrane (Millipore, Billerica, MA, USA), followed by blocking of nonspecific binding sites in 5% nonfat milk in TBST (50 mM Tris-HCl - pH 7.4, 150 mM NaCl, 0.05% Tween 20) for 1 h at room temperature and blotted with primary antibodies in TBST overnight at 4°C. The following antibodies were used: anti-phospho-RIPK1 (Ser166) (Cell Signaling # 31122S), anti-RIPK3 (D8J3 L) (Cell Signaling # 15828S), anti-MLKL (D2I6N) (Cell Signaling #14993S), anti-phospho-MLKL (Cell Signaling #91689S) and anti-β-actin (Sigma, #A1978). Proteins of interest were identified by incubating the membrane with IRDye^®^ LICOR secondary antibodies in TBST, followed by fluorescence imaging detection using the Odyssey^®^ system (CLx Imaging System). Protein bands were quantified by densitometric image analysis using ImageJ software. All the data were normalized by β-actin expression quantification.

### Statistical analysis

All experiments were carried out at least three times independently, including technical replicates in each assay. Statistical analysis was carried out using GraphPad Prism software. *P* values were calculated by unpaired Student’s t test, except for PMC calculated with Wilcoxon rank-sum test. The results are expressed as the mean ± SEM (median (IQR)). The significance of the survival curves was evaluated using the log-rank (Mantel−Cox) test. *P* values < 0.05 were considered statistically significant.

## Results

### IAV and HA induce inflammatory programmed cell death in primary macrophages

**Figure 1:**
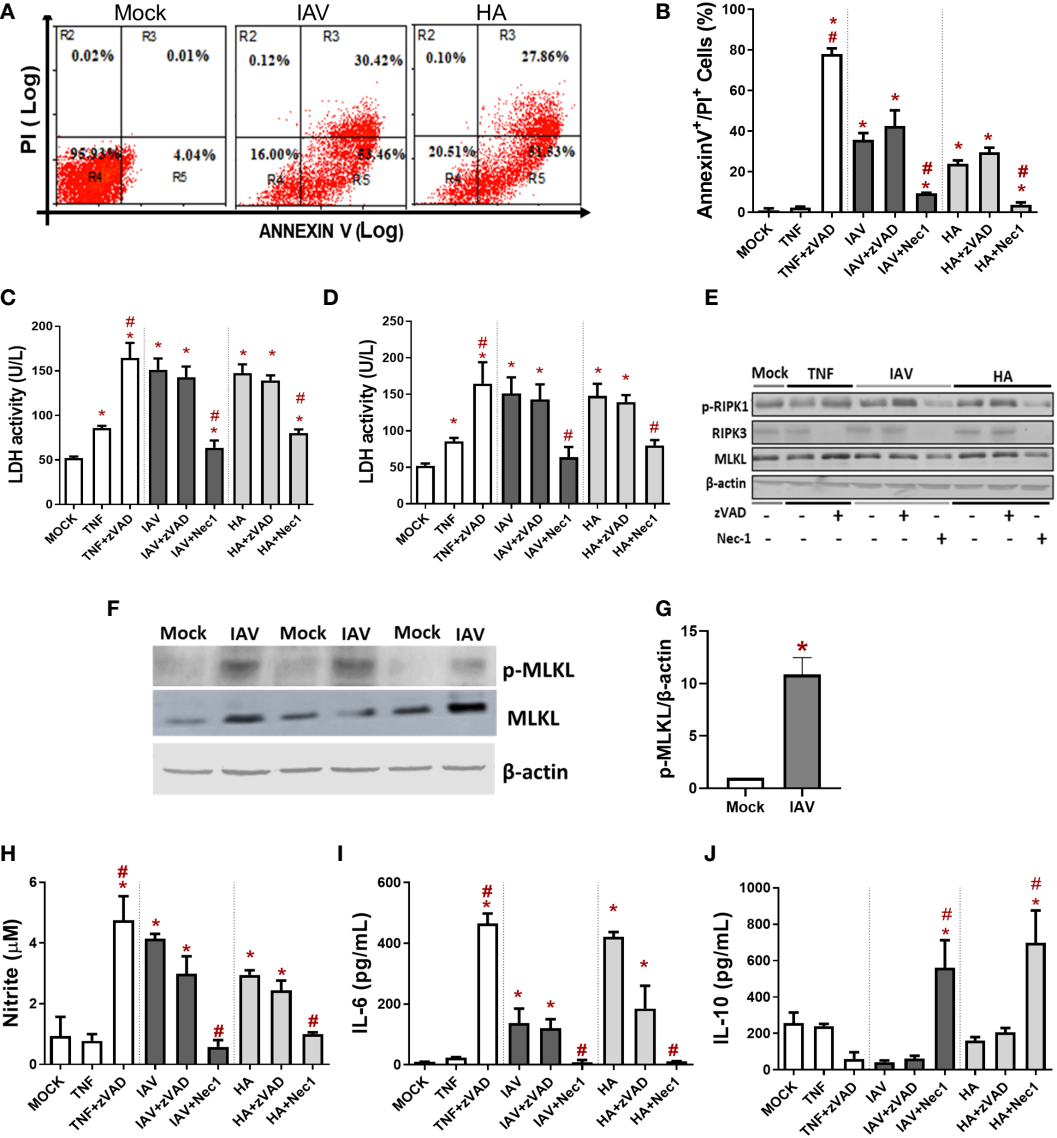
IAV and viral HA induce inflammatory programmed cell death in primary macrophages. Murine (BMDM) or human macrophage (MDM) cultures were pretreated with zVAD (10 µM) or Nec-1 (25 µM), infected with IAV at an MOI of 0.25, and exposed to 10 ng/mL HA or 1 ng/mL TNF-α. **(A, B)** BMDM cell death was also evaluated by flow cytometry analysis of Annexin V/PI-positive cells. Assessment of cell viability through the measurement of LDH release in the supernatant of BMDMs **(C)** or humans **(D)**. **(E)** The expression of p-RIPK1, RIPK3 and MLKL was detected in BMDM lysates by Western blotting. β-actin levels were used as a control for protein loading. **(F)** The expression of p-MLKL was detected in BMDMs by Western blotting. β-actin levels were used as a control for protein loading. **(G)** Graphs of band densitometry of MLKL and p-MLKL obtained after loading normalization and expressed as fold change over mock control. Data are presented as the mean ± SEM of 3 independent experiments ^*^ *P* < 0.05 *versus* the control group (MOCK); **(H)** Levels of nitrite were measured by the Griess method in the supernatant of BMDM cultures after 24 h of stimulus. The levels of **(I)** IL-6 and **(J)** IL-10 were measured by ELISA in the supernatant of BMDM cultures after 24 h of stimulation. Data are presented as the mean ± SEM of 5 independent experiments *P < 0.05 versus control group (MOCK); ^#^P < 0.05 versus the respective untreated infected/stimulated group.

For initial assessments of IAV-induced macrophage death, we evaluated the cell surface exposure of phosphatidylserine (PS) by AnnexinV labeling and the loss of plasma membrane integrity by PI labeling through flow cytometry. We observed that IAV increased the number of both AnnexinV^+^ and AnnexinV^+^/PI^+^ cells (**Figure 1A**). Because the IAV life cycle in macrophages is generally the dead end (Marvin et al., 2017), we tested whether the viral protein responsible for initial attachment, HA, could also trigger a similar cell fate. Indeed, HA also enhanced the number of AnnexinV^+^ and AnnexinV^+^/PI^+^ macrophages (**Figure 1A**). Next, we treated murine macrophages with pharmacological inhibitors of necroptosis (Nec-1, an inhibitor of RIPK1) or apoptosis (zVAD, a pancaspase inhibitor) before IAV infection. Nec-1 pretreatment prevented the increase in annexin V+/PI+ cellular content (**Figures 1B**, **S3**) and LDH release quantified in the culture supernatant (**Figure 1C**), whereas zVAD was not able to prevent these events. The same results were obtained with human macrophages because Nec-1 prevented IAV- and HA-induced LDH release (**Figure 1D**). Of note, TNF was used as a positive control to induce apoptosis (Dhuriya and Sharma, 2018), and TNF/zVAD was used as a positive control of necroptosis (Wu et al., 2011). Consistent with cytometry findings and LDH release, IAV and HA significantly increased the expression of p-RIPK1/RIPK3, which transduces the necroptotic signal to the effector protein mixed lineage kinase domain-like (MLKL) (**Figures 1E**, **S4A–C**), the pore-forming protein involved in membrane disruption (Dhuriya and Sharma, 2018; Nailwal and Chan, 2019). Pretreatment with Nec-1 reduced the levels of p-RIK1, RIPK3, and MLKL and prevented IAV- and HA-induced engagement of necroptotic signals in BMDMs (**Figures 1E**, **S4**). Moreover, we observed that IAV infection increased the phosphorylation of MLKL, the activated form of MLKL that causes plasma membrane rupture (**Figures 1F, G**). These results suggested IAV-induced necroptosis in macrophages. However, the validation of necroptosis induction by IAV infection needs to be supported by additional studies using the MLKL inhibitor necrosulfonamide in macrophages.

Macrophages infected with IAV or exposed to HA produced nitric oxide (**Figure 1H**) and IL-6 (**Figure 1I**). Nec-1 pretreatment of IAV/HA-exposed cells completely shifted the macrophage phenotype: preventing virus-induced enhancement of nitric oxide (**Figure 1H**) and IL-6 (**Figure 1I**) levels while increasing the content of the regulatory cytokine IL-10 (**Figure 1J**). Together, these data suggest that IAV-induced necroptosis in macrophages may be directly related to the proinflammatory cytokine storm observed in severe disease.

### Toll-like receptor 4 (TLR4) is engaged during IAV- and HA-induced inflammatory programmed cell death

**Figure 2:**
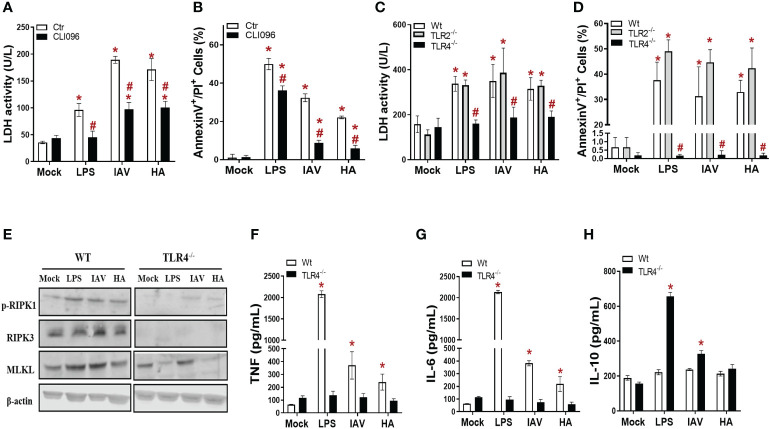
TLR4 engagement is necessary for IAV- and HA-induced inflammatory programmed cell death in macrophages. **(A, B)** BMDM cultures were pretreated with 2 µM CLI95 for 2 h, infected with IAV at an MOI of 0.25, and exposed to 10 ng/mL HA or LPS. After 24 h, LDH was measured **(A)**, and annexin V/PI staining was performed **(B)**. *P < 0.05 versus the respective control group (mock); ^#^P < 0.05 versus the respective untreated infected/stimulated group. **(C, D)** BMDM cultures from WT, TLR2^-/-^ and TLR4^-/-^ mice were infected with IAV at an MOI of 0.25 and exposed to 10 ng/mL HA or LPS. After 24 h, LDH was measured **(C)**, and annexin V/PI staining was performed **(D)**. **(E)** The expression of p-RIPK1, RIPK3 and MLKL was detected in BMDMs by Western blotting. β-actin levels were used as a control for protein loading. The levels of **(F)** IL-6, **(G)** TNF and **(H)** IL-10 were measured by ELISA in the supernatant of BMDM cultures after 24 h of stimulation. ^*^ *P* < 0.05 in comparison to the respective noninfected groups (MOCK) and ^#^ *P* < 0.05 versus the respective Wt infected/stimulated group. The graphs are representative of three independent experiments.

The engagement of TLR4 by HA triggers an innate antiviral and inflammatory response in leukocytes during IAV infection (Liu et al., 2010; Zhang et al., 2015), and we hypothesized that this event could also occur in macrophages as an upstream event to trigger necroptosis. Thus, macrophages were pretreated with a TLR4 signaling pathway inhibitor (CLI95) and exposed to IAV or HA. CLI95-treated IAV/HA-exposed macrophages displayed lower levels of LDH (**Figure 2A**) and annexin V+/PI+ cells (**Figure 2B**) than untreated control macrophages (ctr; **Figure 2**).

Next, pharmacological data were further validated with macrophages from TLR4 knockout mice (TLR4-/-). Of note, macrophages from TLR2 knockout mice (TLR2-/-) were also assayed as a negative control. In the absence of TLR4 signaling, neither IAV nor HA induced lytic cell death above the basal levels, as judged by the similar levels of LDH and annexin V+/PI+ cell counts between TLR4^-/-^ and mock-infected controls (**Figures 2C, D**, **S5**). The absence of TLR2 signaling did not compromise IAV/HA-triggered disruptive cell death in macrophages (**Figures 2C, D**, **S5**).

To gain insight into the role of TLR4 in IAV/HA-triggered necroptosis, we analyzed the expression of p-RIPK1, RIPK3, and MLKL in wild-type (WT) and TLR4^-/-^ macrophages. We observed that the absence of TLR4 impaired the expression of p-RIPK1, RIPK3, and MLKL (**Figure 2E**). Moreover, the lack of TLR4 signaling prevented the increase in the proinflammatory cytokines TNF (**Figure 2F**) and IL-6 (**Figure 2G**) induced by IAV, HA, or LPS (**Figures 2F, G**). On the other hand, the IL-10 levels were enhanced in the TLR4^-/-^ macrophages stimulated with LPS or infected with IAV (**Figure 2H**), suggesting a stronger control of IAV-induced cytokine imbalance *in vitro*.

### IAV or its HA triggers inflammatory programmed cell death is TNF-dependent in macrophages

**Figure 3:**
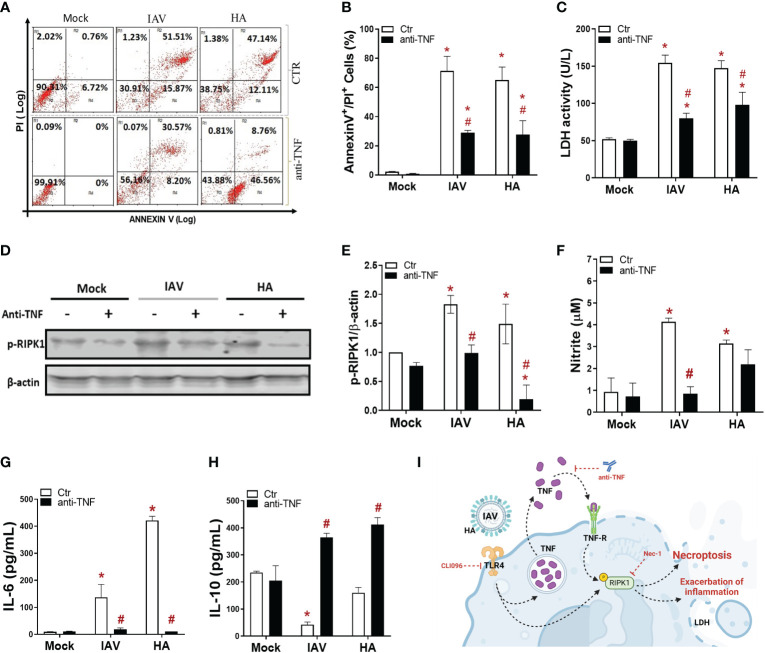
IAV- and HA-induced inflammatory programmed cell death is dependent on TNF. Macrophage cultures were pretreated with 1 ng/mL anti-TNF-α antibody and then infected with IAV (MOI of 0.25) or exposed to 10 ng/mL viral HA for 24 h. Cell death analysis was performed by flow cytometry analysis of annexin V/PI-positive cells **(A, B)**. Assessment of cell viability through the measurement of LDH release in the supernatant of BMDMs **(C)**. **(D)** The expression of p-RIPK1 was detected in BMDMs by Western blotting. β-actin levels were used as a control for protein loading. **(E)** Graphs of band densitometry obtained after loading normalization and expressed as fold change over mock untreated control. **(F)** Levels of nitrite were measured by the Griess method in the supernatant of BMDM cultures after 24 h of stimulus. The levels of **(G)** IL-6 and **(H)** IL-10 were measured by ELISA in the supernatant of BMDM cultures after 24 h of stimulation. Data are presented as the mean ± SEM of 5 independent experiments *P < 0.05 versus untreated control group (MOCK); ^#^P < 0.05 versus respective untreated infected/stimulated group. **(I)** Model of A/HA-triggered necroptosis. IAV/HA triggered necroptosis by a loop of events involving TLR4, TNF-α, and RIPK1, leading to exacerbation of inflammation. Images were created with BioRender.com.

Since IAV/HA induced cell death with the engagement of TLR4 signaling, we next evaluated whether TNF-α, a downstream proinflammatory cytokine produced during these events, could be involved in the necroptotic loop. IAV-infected or HA-exposed macrophages were pretreated with an anti-TNF-α neutralizing antibody, which prevented viral-induced necroptosis by 40-50%, as shown by a reduction in annexin V+/PI+ cell counts (**Figures 3A, B**) and LDH levels (**Figure 3C**). The IAV/HA-enhanced intracellular levels of p-RIPK1 were abolished by anti-TNF-α antibodies (**Figures 3D, E**). By blocking TNF signaling, we also prevented the IAV/HA-induced production of nitric oxide (**Figure 3F**) and IL-6 (**Figure 3G**). Notably, treatment with anti-TNF increased IL-10 levels even in virus-stimulated macrophages (**Figure 3H**). Together, our *in vitro* results suggested that necroptosis induced by IAV/HA is triggered by a loop of events involving TLR4, TNF-α, and p-RIPK1, probably leading to necroptosis and the exacerbation of inflammation (**Figure 3I**).

### Blocking the IAV-induced inflammatory programmed cell death loop ameliorates severely infected animals

**Figure 4:**
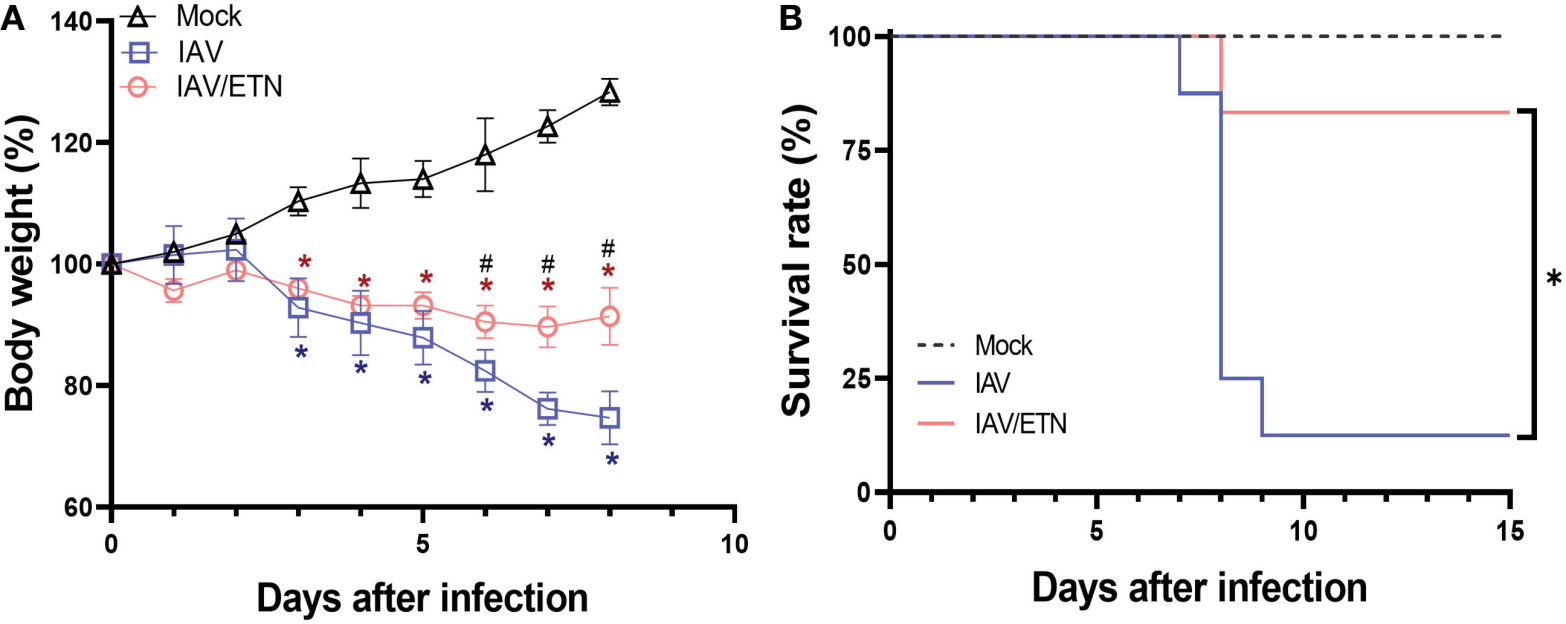
Etanercept reduced weight loss and improved survival during lethal IAV infection in mice. C57Bl/6 mice were inoculated intranasally with 10^3^ PFU of IAV. Six hours postinfection, animals were treated intraperitoneally with 2.5 mg/kg etanercept (ETN). Mice received a daily dose of ETN for 7 days and were monitored for survival **(A)** and body-weight loss **(B)** analysis. Data are shown as the percentage of survival and weight. The graphs are representative of three independent experiments. ^*^ *P* < 0.05 in comparison to the IAV-infected untreated group; ^#^P < 0.05 in comparison to Mock group.

Our next step was to assess whether clinically approved etanercept/ENBREL (anti-TNF-α biodrug) could impair the IAV-induced necroptotic loop. During the *in vivo* experiment, we observed that IAV led to significant weight loss on the 3rd day post-infection (DPI) (**Figure 4A**). In contrast, etanercept (ETN) treatment at a reference dose (2.5 mg/kg) resulted in weight stabilization of IAV-infected mice, showing a significant difference compared to untreated mice from 6 DPI onward (**Figure 4A**). The protective effects of etanercept were also observed in survival because IAV-infected etanercept-treated mice presented significantly enhanced survival (by 8-fold) when compared to untreated infected mice (**Figure 4B**).

**Figure 5:**
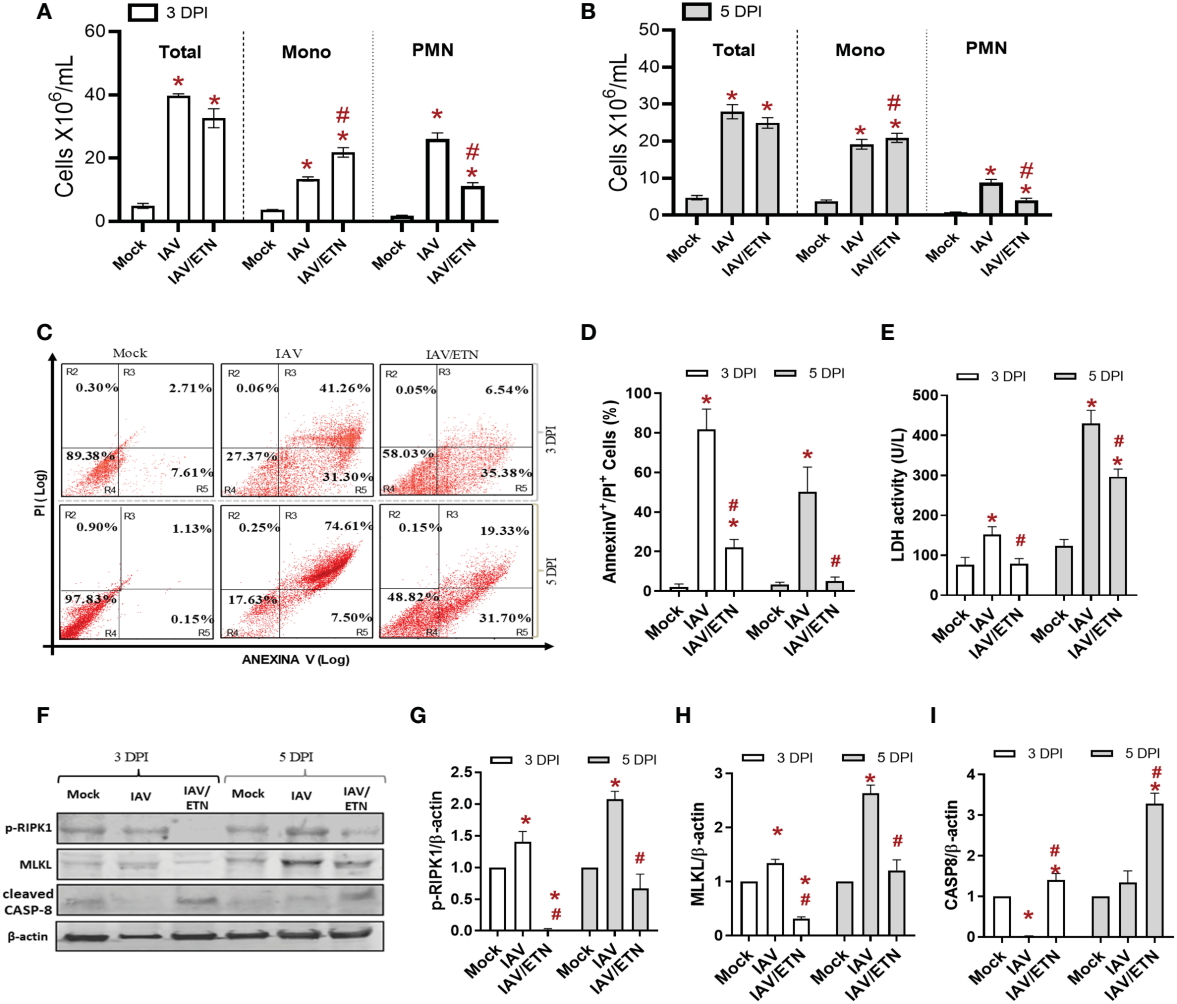
Etanercept reduced inflammatory programmed cell death in macrophages *in vivo*. C57Bl/6 mice were inoculated intranasally with 10^3^ PFU of IAV with and without daily treatment with Etanercep (ETN, 2.5 mg/kg, i.p.). At days 3 and 5 postinfection, animals were euthanized, and BAL and lung were collected. **(A, B)** Total and differential cell counts in bronchoalveolar lavage (BAL) are represented as the number of differential cell counts at days 3 **(A)** and 5 **(B)** postinfection. Total: leukocytes total, Mono: mononuclear leukocytes, PMN: polymorphonuclear leukocytes. **(C, D)** Mononuclear cell death was evaluated by annexin V/PI labeling. **(E)** Lung damage was evaluated by measuring LDH release in the bronchoalveolar lavage (BAL); **(F–I)** The expression of p-RIPK1, MLKL, and cleaved Caspase-8 was detected in the lung tissue homogenates by Western blotting. β-actin levels were used as a control for protein loading. **(G–I)** Graphs of band densitometry obtained after loading normalization and expressed as fold change over mock control. Data are expressed as the means ± SEMs; one-way ANOVA with Dunnett’s *post hoc* test. ^*^ *P* < 0.05 in comparison to mock and ^#^ *P* < 0.05 in comparison to the IAV-infected untreated group. Experiments were performed with 4-6 mice/group.

We next analyzed the respiratory tract of the mice at 3 DPI and 5 DPI before the mortality peak. Despite not inhibiting total leukocyte accumulation in BAL fluid (**Figures 5A, B**), etanercept treatment significantly reduced the number of polymorphonuclear leukocytes (PMNs) at both times analyzed (**Figures 5A, B**). Moreover, the etanercept-treated group presented a significant increase in mononuclear leukocytes at 3 DPI. We observed that etanercept reduced the content of annexin V+/PI+ mononuclear cells induced by IAV, suggesting a reduction of inflammatory programmed cell death during both times analyzed (**Figures 5C, D**). Moreover, the etanercept-treated group also presented a reduction in IAV-induced overall cell death levels, as measured by LDH levels in BAL (**Figure 5E**). In this context, we also evaluated whether the TNF pathway blocking strategy could also reduce necroptosis in lung tissue (**Figure 5F**). We observed an increase in the expression of pRIPK1 and MLKL in the lung tissue of IAV-infected mice, whereas the maximum occurred at 5 DPI. Treatment with etanercept prevented the increase in these proteins (**Figures 5G, H**) but also led to an increase in cleaved caspase-8 (**Figure 5I**), a crucial initiator of the death receptor-mediated apoptotic pathway.

**Figure 6:**
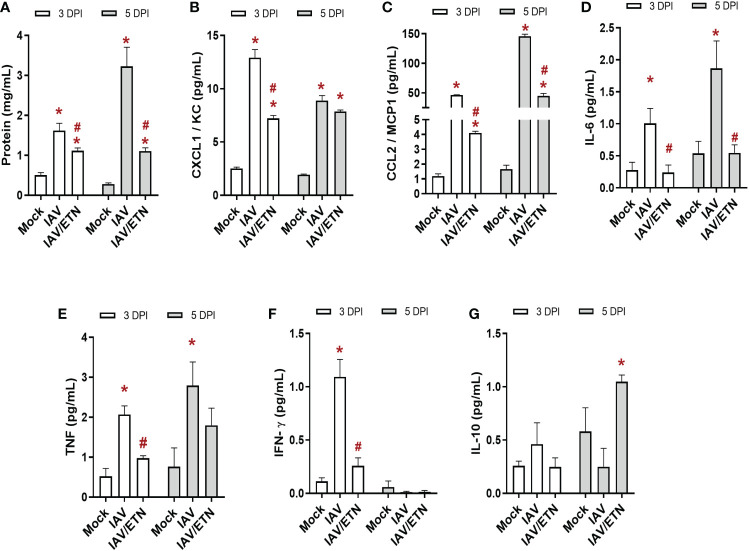
Etanercept reduced the *cytokine storm* in IAV-infected mice. C57Bl/6 mice were inoculated intranasally with 10^3^ PFU of IAV with and without daily treatment with Etanercep (ETN, 2.5 mg/kg, i.p.). At days 3 and 5 postinfection (DPI), animals were euthanized, and bronchoalveolar lavage (BAL) was collected for quantification of total protein **(A)** and inflammatory mediators. The levels of CXCL1/KC **(B)**, CCL2/MCP1 **(C)**, IL-6 **(D)**, TNF **(E)**, INF-γ **(F)** and IL-10 **(G)** were measured by ELISA. Data are expressed as the means ± SEMs; one-way ANOVA with Dunnett’s *post hoc* test. ^*^ *P* < 0.05 in comparison to mock and ^#^ *P* < 0.05 in comparison to the IAV-infected untreated group. Experiments were performed with 4-6 mice/group.

We also evaluated the effects of etanercept on lung inflammation induced by IAV infection. First, we quantified the level of proteins present in BAL, an important biomarker of pulmonary edema (**Figure 6A**). We observed that etanercept prevented the increase in proteins in BAL at both analyzed times. Regarding the main chemokines involved in leukocyte recruitment, we observed that etanercept reduced CXCL1/KC (**Figure 6B**) and CCL2/MCP1 (**Figure 6C**) levels at 3 DPI and 5 DPI. Concerning the proinflammatory and regulatory cytokine responses, pretreatment with etanercept also significantly reduced IL-6 (**Figure 6D**) and TNF levels (**Figure 6E**) induced by IAV infection in the BAL. Notably, we observed that the treated group presented a reduction in IFN-γ levels early (**Figure 6F**) and an increase in IL-10 levels at 5 DPI (**Figure 6G**). Together, our data suggest that the impairment of a necroptosis loop ameliorates cytokine storms induced by IAV infection.

## Discussion

Emerging and re-emerging IAV strains are responsible for seasonal epidemics and pandemics, leading influenza to be a continuous warning to global public health systems (Iuliano et al., 2018; World Health Organization, 2019). One of the biggest challenges in the development of influenza antivirals is the difficulty of providing early treatments during acute viral infections to improve clinical outcomes (Gu et al., 2021). Host-directed broad-spectrum antimicrobial drugs have been used against respiratory viral infections, such as influenza and coronavirus disease 2019 (COVID-19) (Li et al., 2019; Pan et al., 2021). In this context, the use of immunomodulatory drugs has been reported as a promising approach for treating hypercytokinemia induced by acute viral infections (Gu et al., 2021).

After viral infection, an innate response is triggered in host cells to produce interferons, cytokines and chemokines to lead to immune activation, and in certain circumstances, the final fate of the host cell is its death (Orzalli and Kagan, 2017). The various ways by which cells die may directly influence viral physiopathology and patient clinical outcomes (Yatim and Albert, 2011; Nailwal and Chan, 2019). With respect to pulmonary viral infections, it has been reported that different viruses induce specific types of death mechanisms. Recently, we reported that pyroptosis is triggered by SARS-CoV-2 in human monocytes, either by experimental infection or in critically ill COVID-19 patients (Ferreira et al., 2021). For IAV, necroptosis and apoptosis have been reported in epithelial airway cells (Kuriakose et al., 2016; Nogusa et al., 2016; Thapa et al., 2016; Zhang et al., 2020). Here, we further described that macrophages exposed to IAV or just its surface glycoprotein HA experience inflammatory programmed cell death, which leads to an imbalance of proinflammatory and regulatory modulators associated with cytokine storms and severe influenza *in vitro* and *in vivo*.

Indeed, necroptosis has been described as a highly inflammatory process (Dhuriya and Sharma, 2018), leading to lung dysfunction and the development of severe multiorgan tissue damage during virus infections (de Jong et al., 2006; Fajgenbaum and June, 2020; McGonagle et al., 2020). Importantly, the inhibition of necroptosis in IAV/HA-exposed cells shifted macrophages from a pro-inflammatory to a regulatory phenotype (O’Neill et al., 2016), because IL-6 and nitric oxide levels were reduced, while IL-10 levels were enhanced. Despite our results suggesting that necroptosis is the probable cell death induced by IAV infection in macrophages, other lytic cell death pathways may also be triggered in parallel, such as late apoptosis, pyroptosis, and ferroptosis (Nailwal and Chan, 2019; Martens et al., 2021; Morgan and Kim, 2022). Moreover, it is crucial to highlight that Necrostatin-1 is a specific inhibitor of RIPK1. Despite being an important protein in necroptosis pathways, RIPK1 is also involved in multiple regulated cell death and pro-inflammatory pathways (Bertheloot et al., 2021). Additionally, both RIPK3 and MLKL participated in cell activation, cell signaling, and in multiple cell death pathways (Nailwal and Chan, 2019; Martens et al., 2021; Morgan and Kim, 2022).

We observed that TLR4, which plays a major role in the recognition of PAMPs (He et al., 2011; Kaiser et al., 2013; Bertheloot et al., 2021), is engaged by IAV and its HA. Following this event, RIPK1 activation, RIPK3 engagement, and p-MLKL expression are hallmarks of the pore-forming event that culminates with cell lysis (Dhuriya and Sharma, 2018),measurable by quantification of PI+ cells and increased LDH levels detected throughout this study. Although LPS is the classical TLR4 agonist, several viral glycoproteins can act as PAMPs and engage TLR4, such as Ebola virus glycoprotein (Okumura et al., 2010), Respiratory Syncytial Virus (RSV) F protein (Rallabhandi et al., 2012; Funchal et al., 2015), Vesicular stomatitis virus glycoprotein G (Georgel et al., 2007), the envelope proteins of murine retroviruses (Rassa et al., 2002), SARS-CoV-2 Spike protein (Zhao et al., 2021) and Influenza A virus (IAV) hemagglutinin (HA), in a Myd88-dependent manner (Liu et al., 2010). Moreover, the TLR4/TRIF pathways are central to antiviral immunity against the influenza virus (Shinya et al., 2012; Zhang et al., 2015).

Necroptosis is a TNF-dependent event (Orzalli and Kagan, 2017; Bertheloot et al., 2021) and in fact, this proinflammatory cytokine is a second self-feeding signal crucial in triggering IAV/HA-induced inflammatory programmed cell death in macrophages. The probably necroptotic loop described here when an insult engages RIPK1 and MLKL through engagement of TLRs and TNF receptor (TNFR) signaling in macrophages, has also been suggested by others (He et al., 2011; Kaiser et al., 2013; Nogusa et al., 2016). Moreover, the blockage of TNF by etanercept led to a reduction in the expression of both p-RIPK1 and MLKL in mononuclear cells *ex vivo* while increasing the levels of cleaved caspase 8. Because caspase 8 is related to apoptosis, our data suggest that *in vivo* intervention with etanercept in IAV-infected mice downregulated the necroptotic loop by altering the way monocular cells, especially macrophages, die, allowing the shift from necroptosis to apoptosis (Newton et al., 2019; Someda et al., 2020; Bertheloot et al., 2021) and ultimately enhancing animal survival. On the other hand, the blockade of apoptosis by ZVAD in cotreatment with TLR4 and TLR3 ligands induces necroptosis and cell death autophagy, accompanied by an increase in the expression of interferon-β in macrophages (Chen et al., 2022). These data agree with our findings that show that ZVAD + TNF treatment or IAV infection of macrophages triggers increased pro-inflammatory activation in parallel to necroptosis. However, the contribution of autophagy in necroptosis and inflammation induced by IAV after ZVAD treatment still needs further investigation.

In summary, we demonstrated a positive feedback loop of events that led to necroptosis and exacerbated inflammation in IAV-infected macrophages. Our data demonstrated that IAV infection and HA induced disruptive cell death by engaging TLR4 signaling to generate an enhanced TNF-α level and RIP1K/MLKL activation. Etanercept, which is endowed with anti-TNF-α activity, controlled severe influenza, impairing proinflammatory programmed cell death in IAV-infected mice. The proinflammatory response of etanercept-treated animals changes to a regulatory response, increasing survival. The present work improves the knowledge of influenza pathophysiology by highlighting the importance of macrophage cell death during severe infection.

## Data availability statement

The original contributions presented in the study are included in the article/**Supplementary Material**. Further inquiries can be directed to the corresponding author.

## Ethics statement

The studies involving human participants were reviewed and approved by Institutional Review Board (IRB) of the Oswaldo Cruz Institute/Fiocruz (Rio de Janeiro, RJ, Brazil) under the number 49971421.8.0000.5248. The patients/participants provided their written informed consent to participate in this study. The animal study was reviewed and approved by Committee on the Use of Laboratory Animals of the Oswaldo Cruz Foundation, license number L050/15.

## Author contributions

AF and TS conceptualized the study. AF, CS, and FP-D were equally responsible for design and conducted the experiments, literature review, prepared the figures and tables. The manuscript was written by AF, CS, FP-D, and TS, and edited by all authors. CS and NF-R performed virus isolation and titration. CS, NF-R, CF, AM, GM, and MC contributed to the *in vitro* data collection and analysis. AF, FP-D, MM, IA-Q, AC, and JE performed *in vivo* treatment and infection. CG, PB, FB, and TS contributed to study design and critically revised the article. All authors contributed to the article and approved the submitted version.
