# Supplementary material for: Severe influenza infection is associated with inflammatory programmed cell death in infected macrophages
Source: Front Cell Infect Microbiol. 2023 Feb 16;13:1067285. doi: 10.3389/fcimb.2023.1067285 (PMC9980436; doi:10.3389/fcimb.2023.1067285)
Supplement: Supplementary file 1 [file DataSheet_1.docx]

**Severe influenza infection is associated with inflammatory programmed cell death in infected macrophages.**

André C. Ferreira^1,2,3,*^, Carolina Q. Sacramento^1,2,*^, Filipe S. Pereira-Dutra^1,*^, Natália Fintelman-Rodrigues^1,2^, Priscila P. Silva^1,3^, Mayara Mattos^1,2^, Caroline S. de Freitas^1,2^, Andressa Marttorelli^1,2^, Gabrielle R. de Melo^1^, Mariana C. Macedo^1^, Isaclaudia G. Azevedo-Quintanilha^1^, Aluana S. Carlos^3^, João Vítor Emídio^3^, Cristiana C. Garcia^4^, Patrícia T. Bozza^1^, Fernando A. Bozza^1,5,6,#^ and Thiago M. L. Souza^1,2,#,$^

**Affiliation**

^1^ Laboratory of Immunopharmacology, Oswaldo Cruz Institute, FIOCRUZ, Rio de Janeiro, RJ, Brazil

^2^ National Institute for Science and Technology on Innovation on Neglected Diseases (INCT/IDN), Center for Technological Development in Health (CDTS), FIOCRUZ, Rio de Janeiro, RJ, Brazil;

^3^ Preclinical Research Laboratory, Universidade Iguaçu (UNIG), Nova Iguaçu, RJ, Brazil;

^4^ Respiratory and Measles Virus Laboratory, IOC, Fiocruz, Rio de Janeiro, RJ, Brazil.

^5^ National Institute of Infectious Disease Evandro Chagas, FIOCRUZ, Rio de Janeiro, RJ, Brazil.

^6^ Department of Critical Care, Instituto D'Or de Pesquisa e Ensino (IDOR), Rio de Janeiro, RJ, Brasil.

^*^ The authors contributed equally as the first authors.

# The authors contributed equally as the last authors.

^$^ *Corresponding author*: tmoreno@cdts.fiocruz.br

**Keywords:** Influenza virus, , TNF, etanercept, Macrophages, Necroptosis


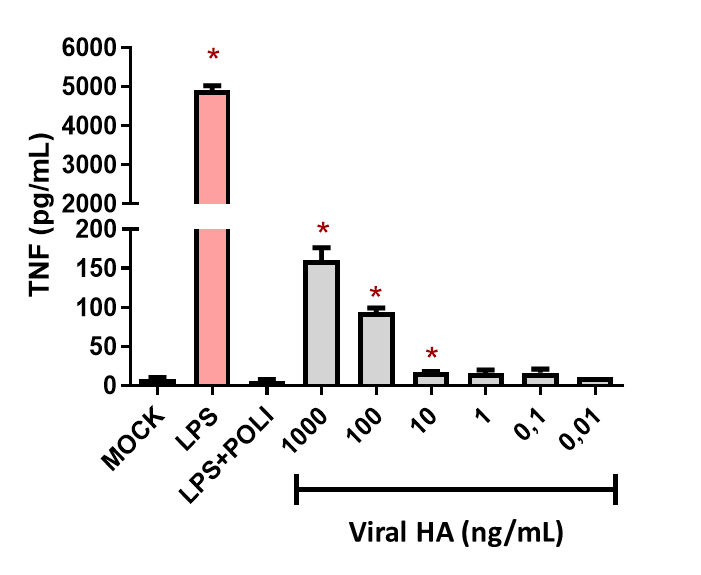


**Figure S1. Type 1 HA from IAV enhances TNF levels in a dose-dependent manner**. Murine macrophages were pretreated with 100 ng/mL POLI for 2 h and exposed to different concentrations of HA or 10 ng/mL LPS. After 24 h, culture supernatants were collected, and TNF-α levels were quantified by ELISA. ^*^ *P* < 0.05 in relation to the control group (MOCK).


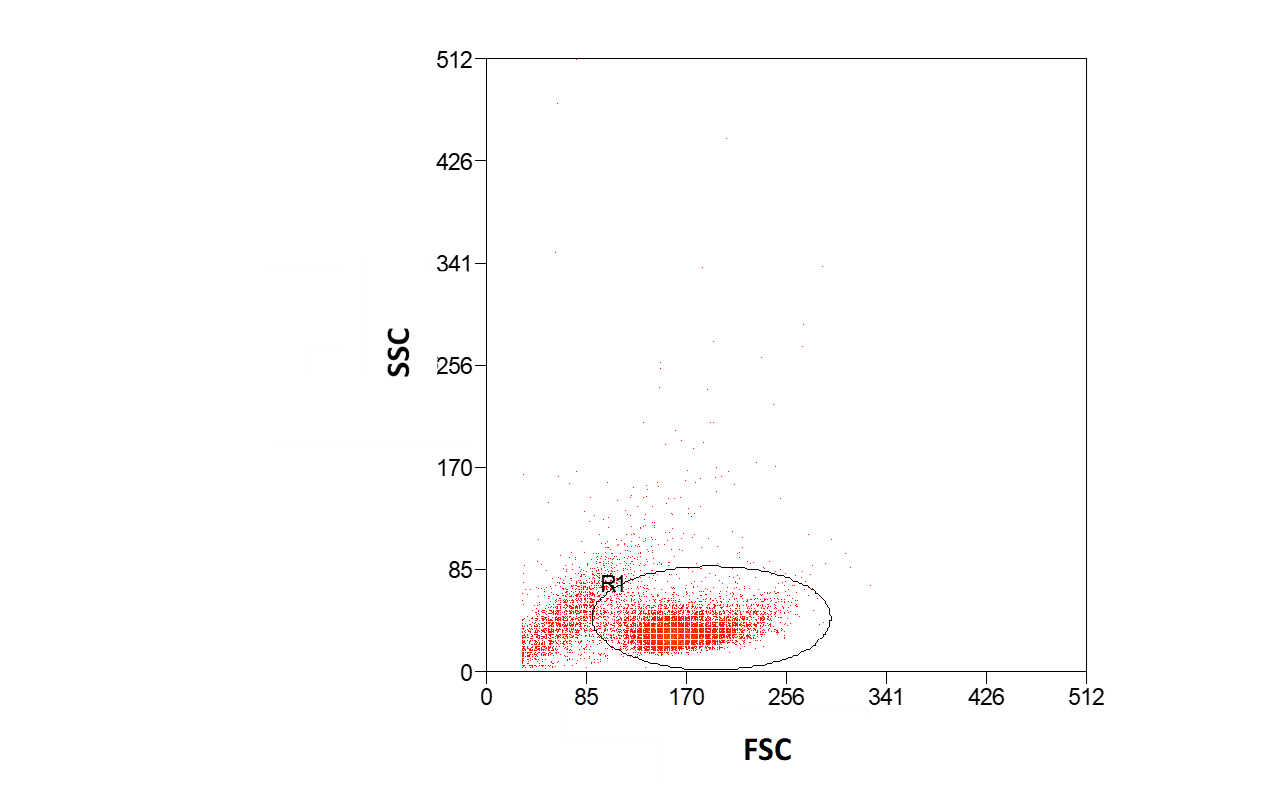


**Figure S2. Flow cytometry gating strategy to select macrophages.** Macrophage monolayers were harvested from *in vitro* experiments and washed twice with cold PBS, and the cell suspension was adjusted to a density of 1 x 10^5^ cells/mL. The cell suspension was analyzed by flow cytometry. Approximately 10,000 events were acquired using a FACSCalibur, and analysis was performed using CellQuest software. Macrophages were gated through cell size (FSC) and granularity (SSC) analysis (circled area). Dot plots are representative of three different experiments.


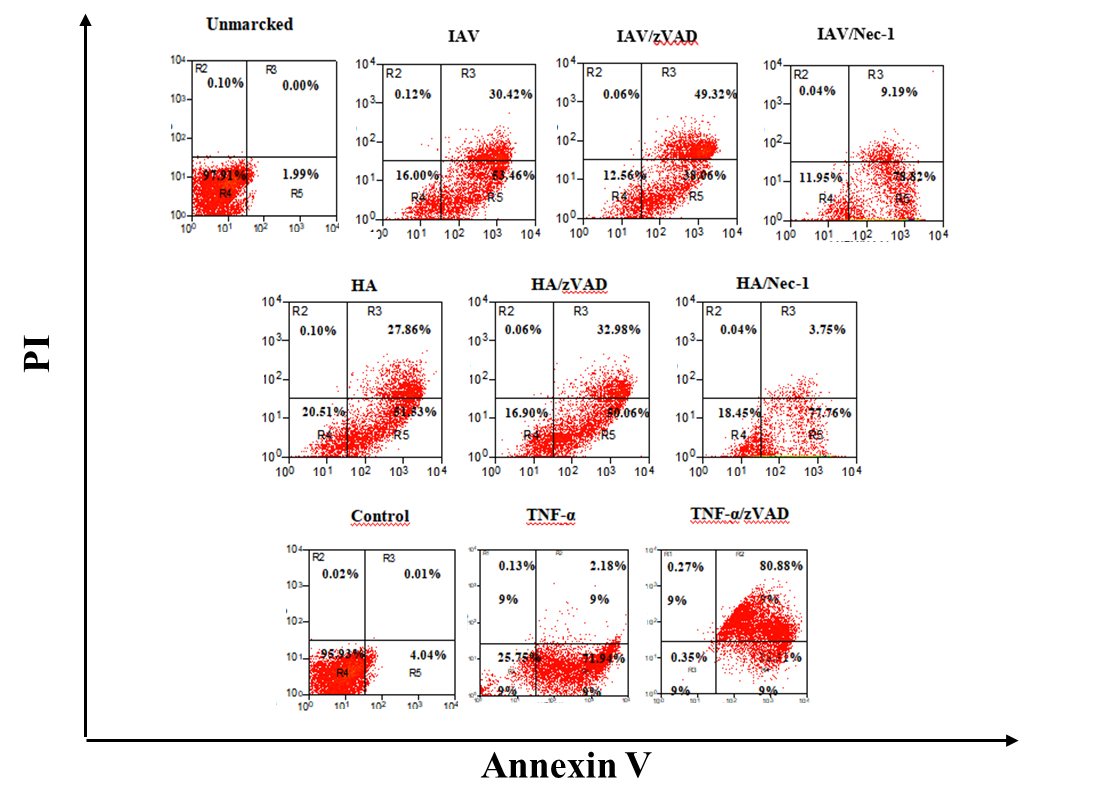


**Figure S3. Representative dot plots of cell death analysis by flow cytometry.** Murine was pretreated with the different pharmacological inhibitors zVAD (10 µM) or Nec-1 (25 µM) for 2 h and then infected with IAV at an MOI of 0.25 (upper panels) or exposed to 10 ng/mL viral HA (middle panels). In parallel, as a positive control, macrophage cultures were also exposed to 1 ng/mL TNF-α (lower panels). After 24 h, the cell monolayers were harvested and labeled with Annexin V and PI for cell death evaluation by flow cytometry. Macrophages were gated through cell size and granularity analysis (Figure S2). This dot plot is representative of the gate strategy to identify the different cellular populations with respect to AnnexinV and PI labeling. At least three independent experiments were performed for each assay in which we described Annexin V and PI labeling.


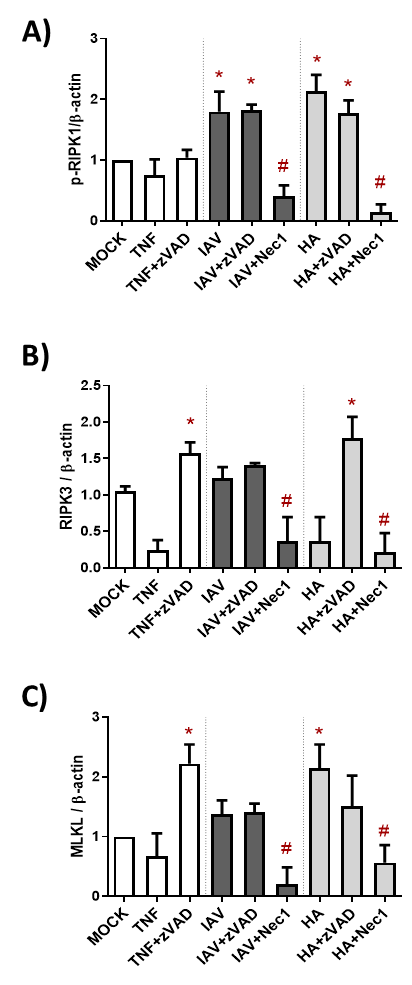


**Figure S4: IAV infection increased the expression of p-RIK1 and p-MLKL in macrophages (A-C)** Graphs of band densitometry of p-RIPK1, RIPK3 and MLKL obtained after loading normalization and expressed as fold change over mock control. Data are presented as the mean ± SEM of 5 independent experiments ^*^ *P* < 0.05 *versus* the control group (MOCK); # P < 0.05 versus the respective untreated infected/stimulated group. β-actin levels were used as a control for protein loading.


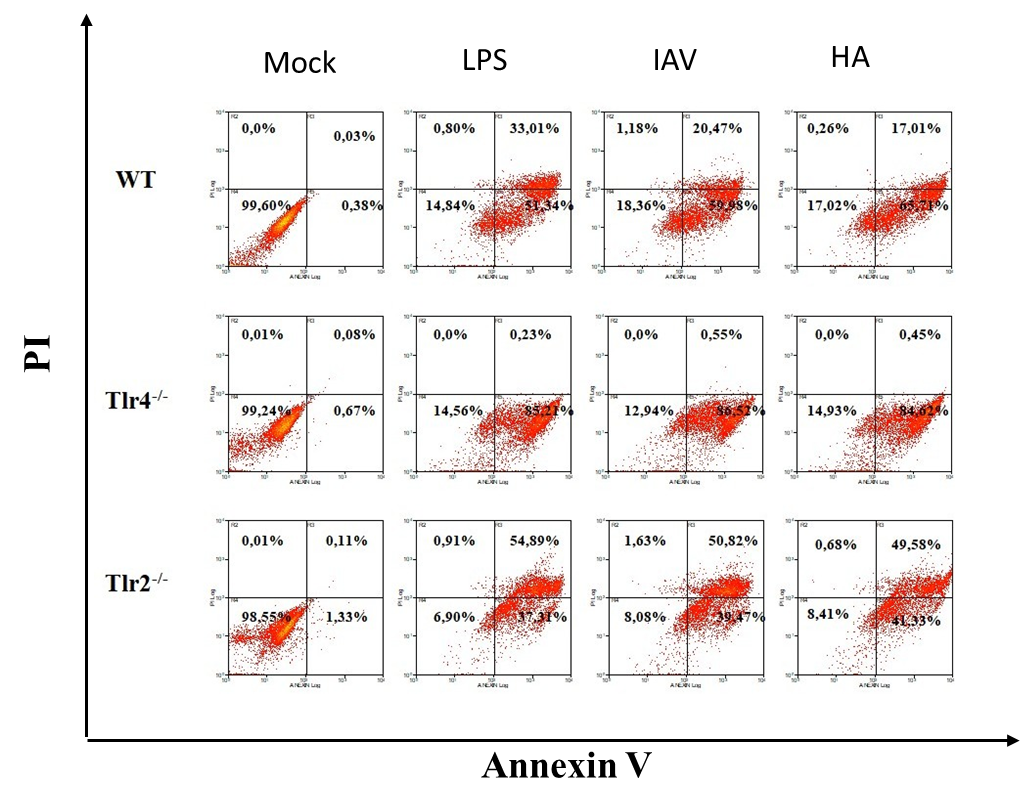


**Fig. S5. TLR4 engagement is necessary for IAV- and HA-induced inflammatory programmed cell death in infected macrophages.** Analysis of cell death by flow cytometry by Annexin V+/PI+, where murine macrophages WT, TLR4^-/-^ or TLR2^-/-^ were infected with influenza virus or treated with HA. Dot plots are representative of three experiments.
